# A Novel Model of Venovenous Extracorporeal Membrane Oxygenation in Rats with Femoral Cannulation and Insights into Hemodynamic Changes

**DOI:** 10.3390/biomedicines12081819

**Published:** 2024-08-10

**Authors:** Fabian Edinger, Thomas Zajonz, Nico Mayer, Götz Schmidt, Emmanuel Schneck, Michael Sander, Christian Koch

**Affiliations:** Department of Anesthesiology, Operative Intensive Care Medicine and Pain Therapy, University Hospital, Justus-Liebig-University, 35392 Giessen, Germany

**Keywords:** recirculation, VV ECMO, inflammation

## Abstract

The application of venovenous (VV) extracorporeal membrane oxygenation (ECMO) has gained wide acceptance for the treatment of acute severe respiratory failure. Since no rat model of VV ECMO therapy with femoral drainage has yet been described, although this cannulation strategy is commonly used in humans, this study aimed to establish such a model. Twenty male Lewis rats were randomly assigned to receive a sham procedure or VV ECMO therapy. After the inhalative induction of anesthesia, animals were intubated and the vascular accesses were placed surgically. While venous drainage was achieved through a modified multi-orifice 18 G cannula that was placed in the inferior vena cava through the femoral vein over a guide wire with an ultra-flexible tip, the venous return was realized via a shortened 20 G cannula into the jugular vein. Hemodynamic data were obtained from a tail artery and left ventricular pressure–volume catheter. Repetitive blood gas analyses were carried out, and systemic inflammation was measured using an enzyme-linked immunosorbent assay. While animals in the ECMO group showed adequate oxygenation and decarboxylation, there was no evidence of recirculation. VV ECMO therapy increased stroke volume (SV), cardiac output (CO), and left ventricular end-diastolic volume (LVEDV). ECMO-induced inflammation was reflected in increased levels of tumor necrosis factor alpha. However, no differences in interleukins 6 and 10 were seen. This study describes a frequently used cannulation strategy in humans for a rat model of VV ECMO. Despite successful oxygenation and decarboxylation, the oxygenated blood may reduce pulmonary vascular resistance and lead to an increased LVEDV, which is associated with increased SV and CO. This model allows us to answer research questions about topics such as intestinal microcirculation in further studies.

## 1. Introduction

While the first use of a cardiopulmonary bypass was described in 1953, the first prolonged use of venovenous (VV) extracorporeal membrane oxygenation (ECMO) therapy in a patient with acute respiratory distress syndrome (ARDS) was published in 1972 [1,2]. The worldwide use of VV ECMO therapy has increased tremendously over the last 15 years for several reasons. First, in 2009, the conventional versus extracorporeal membrane oxygenation for severe adult respiratory failure (CESAR) trial was published, reporting a positive treatment effect of VV ECMO compared to conventional therapy in adults with life-threatening ARDS [3]. Second, the 2009 influenza pandemic, which caused an increase in ARDS cases, led to the increased use of VV ECMO treatment [4]. Third, the severe acute respiratory syndrome coronavirus-2 pandemic has been associated with an increased demand for VV ECMO therapy [5].

However, ECMO therapy can be associated with significant side effects. The large foreign surface area of the membrane and the circuit leads to contact activation of the inflammatory, coagulation, and complement systems, provoking endothelium activation with the release of proinflammatory cytokines [6]. Bleeding complications or thrombosis of the membrane oxygenator might be life-threatening. In addition, during VV ECMO therapy, highly oxygenated blood is pumped into the central veins. The effect of this non-physiological high-oxygen saturation on the critically ill lung remains unclear. Although randomized controlled clinical trials are the best way to investigate these questions, they have several limitations. A randomized controlled clinical trial with ECMO versus non-ECMO therapy during severe hypoxemia is ethically unacceptable [7]. In addition, critically ill patients with ARDS in the intensive care unit (ICU) represent a heterogeneous collective. Moreover, in the context of multicenter studies, it is difficult to fully standardize patient treatment to generate comparable results. Therefore, large sample sizes are needed for clinical trials.

In this context, animal models offer the advantage of reduced variance through the use of inbred strains. Furthermore, all animals can be treated with exactly the same protocol, which can lead to comparable results. As large animal models are costly and time-consuming, rodent models are preferred. Besides models of veno–arterial (VA) ECMO with jugular drainage and a femoral or carotid return, models of VV ECMO in rats with jugular drainage have been described [8,9,10,11,12,13,14,15,16,17,18,19]. Cho et al. described a rat model with drainage of the right internal jugular vein and a return through the left internal jugular vein [20]. Huang et al. used a double- or three-lumen catheter, which was inserted through the right internal jugular vein. While drainage was realized through the distal lumen, the oxygenated blood was returned through the medial or proximal lumen [18,19,21]. Fujii et al. presented a model with drainage through the right atrium and a return to the femoral vein [17]. However, no rodent model of VV ECMO with femoral drainage has yet been published to our knowledge.

In humans, the percutaneous cannulation of the femoral vein for venous drainage during VV ECMO therapy is favored due to a lower risk profile and reduced recirculation. Therefore, this study aimed to establish a new model of VV ECMO in rats with femoral cannulation to answer research questions in further studies, which are difficult to investigate in humans, like questions about topics such as intestinal microcirculation during VV ECMO therapy.

## 2. Materials and Methods

### 2.1. Animals

All procedures involving living animals were performed in accordance with the standards for animal care and the Animal Research: Reporting of In Vivo Experiments (ARRIVE) guidelines and were authorized by the responsible local committee for animal care (Animal Welfare Commission of the Department of Veterinary Medicine at the Regional Council of Giessen (GI 20/26 Nr. G 77/2019; Regierungspraesidium Giessen, Germany) [22].

Male Lewis rats (330–350 g) purchased from Janvier Labs (Le Genest St. Isle, France) were kept at 22 °C and at 55% relative humidity, using a day/night cycle of 14/10 h, with unlimited access to standard chow and water. The animals were randomly separated into two groups per lot to receive VV ECMO therapy (*n* = 10) or a sham procedure (*n* = 10). During the sham procedure, all cannulas were inserted, and the rats were monitored for 2 h without ECMO support. Contrary to the ECMO group, these animals received no lung-protective ventilation.

### 2.2. Induction and Maintenance of Anesthesia

Following the inhalative induction of anesthesia in an induction chamber (5% isoflurane, Baxter, Unterschleißheim, Germany) balanced with 95% oxygen, rats were intubated endotracheally (16 G cannula, B.Braun, Melsungen, Germany) and ventilated in a volume-controlled and weight-adjusted manner (respiratory rate = 53.3 × body weight [kg]^−0.26^, tidal volume = 6.2 mL × body weight [kg]^1.01^; Harvard Inspira, Harvard Apparatus, Cambridge, UK) with an inspiratory oxygen fraction of 0.5. Thereafter, animals were positioned on an automated heating pad, a rectal temperature probe was inserted, and the temperature was maintained between 36.5 and 37.0 °C. The monitoring consisted of continuous electrocardiogram, measuring heart rate, cardiac output (CO), stroke volume (SV), left ventricular end-diastolic volume (LVEDV) and pressure (LVEDP), left ventricular ejection fraction (LVEF), end-tidal carbon dioxide (MicroCapStar, CWE, Ardmore, PA, USA), and arterial blood pressure (systolic, diastolic, and mean; PowerLab 8/30 signal converter, ADInstruments, Spechbach, Germany). Following endotracheal intubation, the lateral tail vein was percutaneously punctured (24 G cannula, B.Braun, Melsungen, Germany), and a continuous balanced crystalloid infusion was administered at a rate of 5 mL/kg/h (Sterofundin B.Braun, Melsungen, Germany) with midazolam (2 mg/kg/h, Roche, Basel, Switzerland), fentanyl (10 µg/kg/h, Albrecht GmbH, Aulendorf, Germany), and pancuronium (0.1 mg/kg/h, Inresa, Freiburg, Germany).

### 2.3. Cannulation

**Figure 1:**
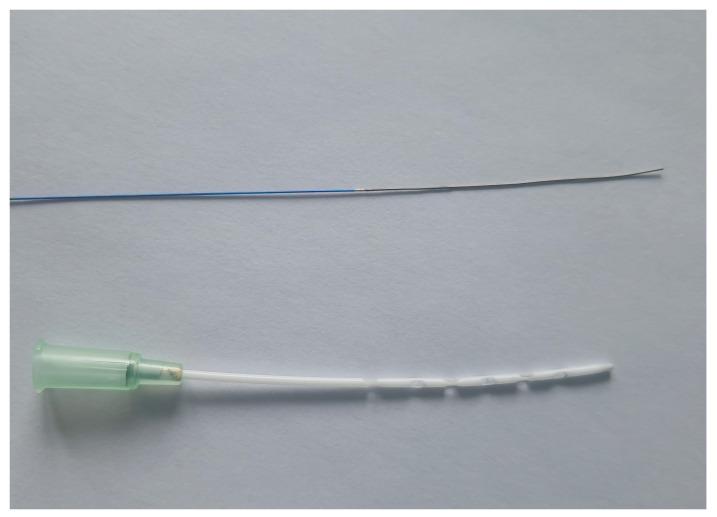
Draining ECMO cannula and ultra-flexible wire. Balanced middle weight wire with ultra-flexible tip (0.36 mm; Abbott, Wetzlar, Germany) and modified draining ECMO cannula with 12 side holes (18 G Surflo, Terumo, Eschborn, Germany) are pictured.

All other vascular accesses were surgically placed. First, the tail artery was cannulated for measurement of the arterial blood pressure and intermittent blood gas analysis (24 G, BBraun, Melsungen, Germany). Next, a small incision was made in the neck, and the right internal jugular vein and carotid artery were dissected. Afterward, a 2 F pressure–volume catheter (SPR-838, Millar, Houston, TX, USA) was inserted through the right carotid artery into the left ventricle for continuous measurement of the left ventricular pressure and volume. Thereafter, the internal jugular vein was cannulated with a modified, shortened 20 G cannula for the ECMO return (Surflo, Terumo, Eschborn, Germany). The right femoral vein was then dissected through a small skin incision and ligated distally. The vessel was first punctured with a modified needle (90° curved tip, Sterican 26 G BBraun, Melsungen, Germany) and a 0.46 mm Seldinger wire (Teleflex Medical GmbH, Fellbach, Germany) was inserted. Afterward, a 24 G cannula (B.Braun, Melsungen, Germany) was inserted into the vessel over the wire as an introducer for the next Seldinger wire (0.36 mm) with an ultra-flexible tip (balanced middle-weight, Abbott, Wetzlar, Germany). This wire was passed across the femoral bifurcation and the introducer was removed. The venous-draining cannula was then inserted into the femoral vein over the wire. The cannula consisted of a modified 64 mm 18 G multi-orifice cannula (18 G Surflo, Terumo, Eschborn, Germany, Figure 1) that was carefully placed completely in the vessel. Before cannulation of the femoral vein, all animals received a bolus of heparin (400 IU/kg, Merckle GmbH, Blaubeuren, Germany) via the lateral tail vein.

### 2.4. Extracorporeal Membrane Oxygenation

As described earlier, the ECMO circuit consisted of a roller pump (Verderflex Vantage 3000, Castleford, UK), a venous reservoir (M. Humbs, Valley, Germany), and a membrane oxygenator (Micro-1, Kewei Rising Medical, Shenzhen, China) [8,9]. While the draining cannula was connected with a shortened Heidelberg extension line (B.Braun, Melsungen, Germany) to the venous reservoir, the membrane oxygenator was linked to the return cannula by a syringe pump line (B.Braun, Melsungen, Germany). A three-way stopcock (B.Braun, Melsungen, Germany) was connected to the venous reservoir for central venous blood gas analysis. The entire circuit was primed with 250 IU of heparin (Ratiopharm, Ulm, Germany) and 9 mL of hydroxyethyl starch 6% (Voluven, Fresenius Kabi, Bad Homburg, Germany). Blood flow started at a rate of 45 mL/kg/min and then gradually increased to 90 mL/kg/min.

Sweep gas flow on the membrane was adjusted to between 20 and 70 mL/min to maintain arterial partial pressure of carbon dioxide (pCO_2_) levels between 35 and 45 mmHg. The oxygen fraction on the ECMO membrane (F_i_O_2mem_) was set to 0.5. Lung-protective ventilation was achieved by adjusting the ventilator during ECMO therapy. The tidal volume and respiratory rate were set to 75% of the rat’s weight according to the formulae: tidal volume = 6.2 mL × body weight (kg)^1.01^ and respiratory rate = 53.3 × body weight (kg)^−0.26^.

### 2.5. Timepoints of Hemodynamic Measurements

Baseline values were recorded before starting ECMO at baseline. Arterial blood pressure and invasive hemodynamic parameters were recorded every 10 min for up to 120 min.

### 2.6. Blood Analyses

Blood samples for blood gas analysis were collected at baseline and every 30 min from the start of the ECMO until 120 min had passed. At each observation point, arterial oxygen saturation (S_a_O_2_), central venous oxygen saturation (S_cv_O_2_), arterial oxygen partial pressure (pO_2_), pCO_2_, hemoglobin, hematocrit, pH, bicarbonate, base excess (BE), lactate, glucose, sodium, potassium, calcium, and chloride were measured (ABL800, Radiometer, Copenhagen, Denmark). In addition, blood samples for inflammation analyses were collected at baseline and every 60 min thereafter until 120 min. This blood was centrifuged at 5000 rpm for 5 min, and the plasma samples were stored directly at −80 °C for further analysis.

### 2.7. End of Experiments

After 120 min, isoflurane was increased to 5.0%, and the animals were euthanized by exsanguination through the draining ECMO cannula.

### 2.8. Enzyme-Linked Immunosorbent Assays

The cytokines tumor necrosis factor alpha (TNF-α), interleukin 6 (IL-6), and interleukin 10 (IL-10) were measured using enzyme-linked immunosorbent assays performed according to the manufacturer’s instructions (ELISA kits R6000B, RTA00, and R1000, R&D Systems, Wiesbaden, Germany). Probes were only thawed once.

### 2.9. Statistics

All data are presented as percentage or medians with 25th and 75th percentiles. Differences between the groups were analyzed using analysis of variance for repeated measurements. The baseline values were not included in the analysis of repeated measurements because the VV ECMO was started just after recording the baseline values.

An inbreed strain was used to reduce the variance between the groups. The alpha and beta errors were set to 0.05 and 0.02, respectively. The use of groups with 10 animals resulted in an effect size of 0.55 (moderate). The calculation was performed using G-Power 3.1.9.2.

A *p*-value of *p* ≤ 0.05 was considered statistically significant. GraphPad Prism version 7 was used for data presentation (GraphPad Software, San Diego, CA, USA) and all statistical analyses were performed using SPSS Version 20 (IBM, Stuttgart, Germany).

## 3. Results

### 3.1. Blood Gas Analysis

**Figure 2:**
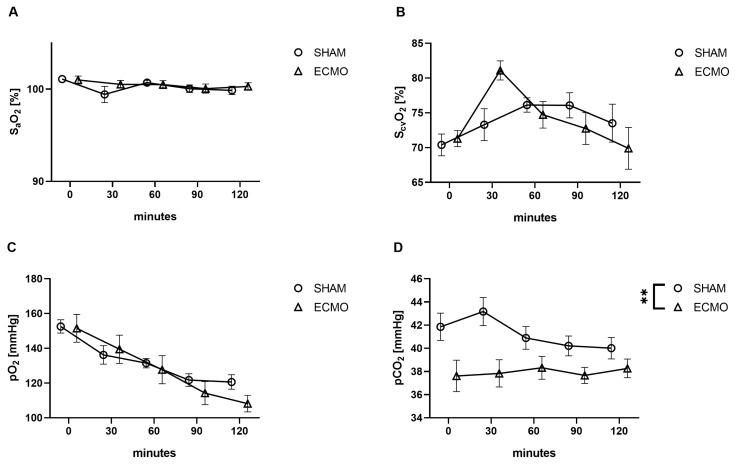
Time course of (**A**) arterial and (**B**) central venous saturation, (**C**) arterial partial pressure of oxygen (pO_2_) and (**D**) carbon dioxide (pCO_2_). While no differences were measured between the sham and VV ECMO groups regarding arterial and central venous saturation and pO_2_, animals in the sham group showed significantly elevated concentrations of pCO_2_. The asterisks denote the degree of statistical significance (** *p* < 0.01). Abbreviations: ECMO = extracorporeal membrane oxygenation; pO_2_ = arterial partial pressure of oxygen; pCO_2_ = arterial partial pressure of carbon dioxide.

No differences were observed between the ECMO and sham groups regarding S_a_O_2_ (*p* = 0.791), S_cv_O_2_ (*p* = 0.992), and pO_2_ (*p* = 0.514; Figure 2). However, a significant increase in pCO_2_ (*p* = 0.007) was noted in animals in the sham group (Figure 2).

During the application of VV ECMO, decreased concentrations of hemoglobin (*p* < 0.001), glucose (*p* = 0.046), and potassium (*p* = 0.001) were measured (Table 1).

Furthermore, increased concentrations of chloride (*p* = 0.002) and lactate (*p* = 0.006) were found in the VV ECMO group (Table 1). The other blood gas analysis results revealed no differences between the two groups and are presented in Table 1.

### 3.2. Hemodynamic Parameters

**Figure 3:**
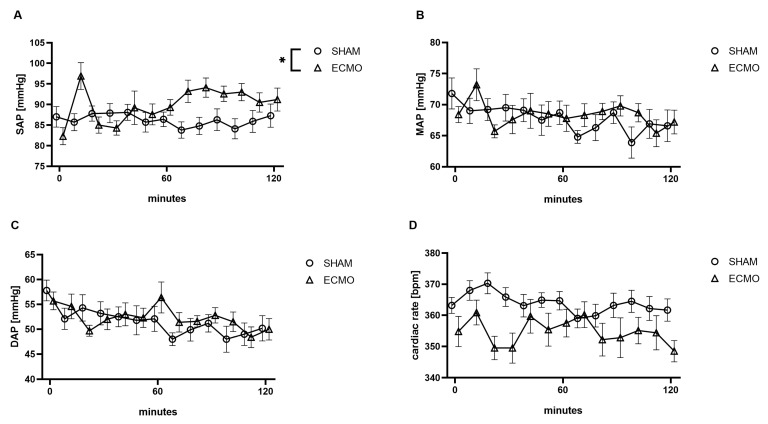
Time course of (**A**) systolic (SAP), (**B**) mean (MAP), (**C**) diastolic arterial blood pressure (DAP), and (**D**) heart rate. During VV ECMO therapy, an elevated SAP was measured compared to sham animals. The asterisks denote the degree of statistical significance (* *p* < 0.05). Abbreviations: DAP = diastolic arterial blood pressure; ECMO = extracorporeal membrane oxygenation; MAP = mean arterial blood pressure; SAP = systolic arterial blood pressure.

While a significantly increased systolic arterial blood pressure (*p* = 0.011) was measured during VV ECMO, no differences were found regarding the mean (*p* = 0.514), diastolic arterial blood pressure (*p* = 0.627), and heart rate (*p* = 0.064; Figure 3).

**Figure 4:**
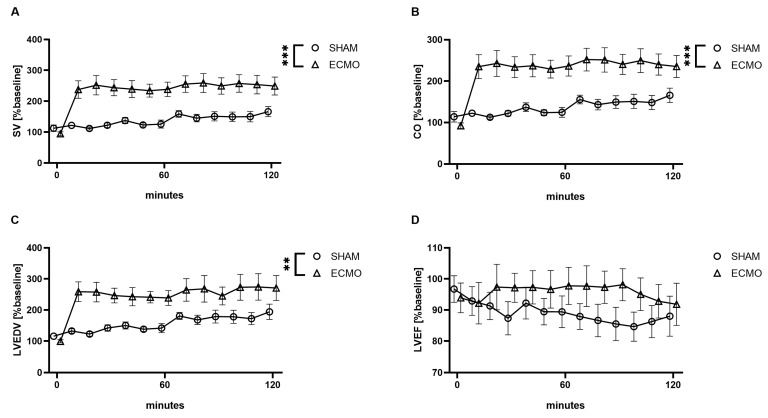
Time course of (**A**) stroke volume (SV), (**B**) cardiac output (CO), (**C**) left ventricular end-diastolic volume (LVEDV), and (**D**) left ventricular ejection fraction (LVEF). During VV ECMO therapy, increased SV, CO, and LVEDV were captured compared to sham animals. Furthermore, no differences were seen regarding LVEF. The asterisks denote the degree of statistical significance (** *p* < 0.01; *** *p* < 0.001). Abbreviations: CO = cardiac output; ECMO = extracorporeal membrane oxygenation; LVEDV = left ventricular end-diastolic volume; LVEF = left ventricular ejection fraction; SV = stroke volume.

Furthermore, analysis of the pressure–volume data revealed an increased SV (*p* < 0.001), CO (*p* < 0.001), and LVEDV (*p* = 0.003) in the VV ECMO group (Figure 4). However, no significant differences were noted regarding LVEDP (*p* = 0.131) and LVEF (*p* = 0.172; Figure 4).

### 3.3. Inflammatory Parameters

**Figure 5:**
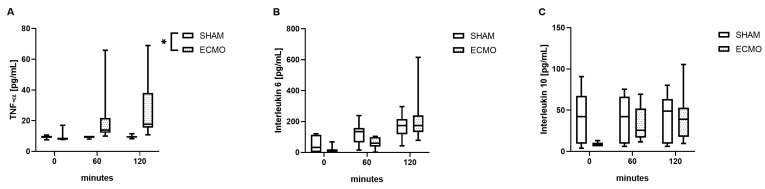
Time course of (**A**) tumor necrosis factor alpha (TNF-α), (**B**) interleukin 6 (IL-6), and (**C**) interleukin 10 (IL-10). During VV ECMO therapy, increased concentrations of TNF-α were measured compared to sham animals. Furthermore, no differences were measured regarding IL-6 and IL-10. The asterisks denote the degree of statistical significance (* *p* < 0.05). Box and whisker plots indicate the median, interquartile range (box), and minimum and maximum (whiskers). Abbreviations: ECMO = extracorporeal membrane oxygenation; IL-6 = interleukin 6; IL-10 = interleukin 10; TNF-α = tumor necrosis factor alpha.

During the treatment with VV ECMO, elevated levels of TNF-α (*p* = 0.020) were observed (Figure 4). However, no significant differences were found regarding IL-6 (*p* = 0.851) and IL-10 (*p* = 0.665; Figure 5).

## 4. Discussion

To the best of our knowledge, this is the first description of a model of VV ECMO in rats with femoral drainage and jugular return. In summary, our established model showed adequate oxygenation and decarboxylation during lung-protective ventilation. In addition, elevated systolic blood pressure, SV, and consecutive CO were captured. Furthermore, ECMO-induced inflammation was reflected in elevated serum concentrations of TNF-α.

Several models of VA ECMO therapy in rats have been described [8,10,13,15,23,24]. However, only a few models of VV ECMO therapy in rats have been published [17,19,20]. In the VA setup, drainage of the right atrium allows for adequate blood flow. As oxygenated blood is returned to a central vein during VV ECMO therapy, right atrium drainage is at risk for recirculation of the returned blood fraction [25]. Therefore, cannulation of the femoral vein with drainage of the inferior vena cava is preferred in humans. It should be noted that in the VV ECMO models published by Fujii et al. and Cho et al., the draining cannula was inserted through the jugular vein into the right atrium [17,20]. Therefore, there is a high risk of recirculation of oxygenated blood in these models. Furthermore, it has to be highlighted that only blood flow rates of 50–60 mL/kg/min were used [17,20]. Contrary to this, blood flow rates of 90 mL/kg/min were achieved within our model. Furthermore, Li et al. reported blood flow rates of 80–90 mL/kg/min using a three-lumen 5.5 French central venous catheter as a double lumen cannula with drainage of the inferior cava vein and a return to the right atrium through the internal jugular vein [19]. As the correct positioning of the returning blood flow to the tricuspid valve can be challenging in humans, avoidance of recirculation in rats with a double-lumen cannula appears to be even more complex. From this perspective and for a translational approach, the venous drainage of the inferior vena cava from the femoral vein was preferred in our model. It should be noted that it is very challenging to pass a guide wire through the femoral vein into the inferior vena cava without perforating the vessel. During training, many guide wires perforate the vein in the confluence of the Iliac veins to the inferior vena cava. However, the use of a balanced middle-weight wire with an ultra-flexible tip was a game-changer in this context, and no perforation of the veins was seen during our experiments. As the femoral vessels are smaller than the jugular vein, the diameter of the draining cannula is limited, and therefore, an 18 G cannula was successfully used in this study. To allow sufficient blood flow, a long 64 mm cannula was chosen and prepared with 12 side holes. The correct position of the cannula was confirmed in several rats in an autopsy after the end of the experiment. The unique feature of our model is the use of a guide wire with an ultra-flexible tip, which allows us to place a draining cannula with many side holes in the inferior cava vein through the femoral vein.

Contrary to other rat models of VV ECMO, no differences regarding pO_2_ were measured in our study. It must be considered that Fujii et al. adjusted the F_i_O_2mem_ to 1.0 and the rats were spontaneously breathing room air throughout the experiment [17]. It must also be noted that intubation with controlled ventilation is associated with atelectasis [26]. These facts may explain their reported pO_2_ values being higher than in our study and the significant difference between the ECMO and sham groups [17]. Furthermore, contrary to their experimental setup, our study focused more on a translational approach. Therefore, lung-protective ventilation was applied during VV ECMO therapy. Furthermore, to avoid hyperoxia-induced inflammation, the F_i_O_2mem_ was set to 0.5 and the rats were ventilated with 50% oxygen [11,12]. Despite lung-protective ventilation with reduced tidal volume and respiratory rates, analogous pO_2_ was measured during VV ECMO therapy. In this context, an elevation of the pCO_2_ could be expected due to the reduced alveolar ventilation. The decreased pCO_2_ during VV ECMO therapy in this study underlines that our model performs well. Furthermore, it must be highlighted that during VV ECMO therapy, the oxygenation is accessed by S_a_O_2_ [27]. The fact that the sham group consisted of healthy rats with normal S_a_O_2_ values may explain why no differences from the VV ECMO group were seen. In contrast to other rat models of VV ECMO, S_cv_O_2_ was captured in this study, and the measured values indicated that no relevant recirculation occurred between the returned and drained blood.

Further results of the blood gas analysis revealed decreased concentrations of potassium and elevated levels of chloride, as well as a trend toward increased concentrations of sodium (*p* = 0.052). The reason for these alterations could be the composition of the priming fluid. Besides hydroxyethyl starch, the used Voluven solution consists of sodium and chloride, 154 mmol/L, respectively. Moreover, the decreased concentrations of glucose and hemoglobin could be caused by dilution with the priming volume. The repetitive blood withdrawals further decreased the hemoglobin concentration. Although a significant difference regarding lactate was measured between the sham and VV ECMO groups, the level was 1.2 mmol/L after 2 h of VV ECMO therapy. Therefore, the critical hemoglobin value seems not to have been reached. This assumption is further supported by the measured S_cv_O_2_ at the end of the experiments.

The analysis of the hemodynamic data revealed an elevation in the systolic arterial blood pressure in the VV ECMO group. It must be noted that an initial peak was seen at 10 min. Although rats in the VV ECMO group received a bolus of sedation after ECMO commencement to anticipate dilutional effects, reduced sedation cannot be excluded at this point. While no differences were seen in this study regarding mean arterial blood pressure, Fujii et al. presented a significantly reduced mean arterial blood pressure in animals treated with VV ECMO [17]. It must be noted that a blood flow of only 50 mL/kg/min was used. Furthermore, the reduced mean arterial blood pressure could also reflect recirculation due to the cannulation site in their study (drainage from the right atrium and a return through the femoral vein) [17].

The assessment of CO in humans is challenging during VV ECMO therapy. Thermodilution is affected by the drainage and return of heated blood from the ECMO circuit. Although ultrasound, especially transesophageal echocardiography, allows measurements of the CO, the results are highly dependent on the examiner. From a pathological perspective, the drainage and return of blood from the venous system should not impact the CO. Interestingly, this study demonstrated an increased SV with a consequently elevated CO. To the best of our knowledge, this is the first study describing an elevated CO during VV ECMO therapy. It must be noted that oxygenated blood is flowing through the pulmonary arteries, which could have reduced the pulmonary vascular resistance and led to an increased preload of the left ventricle. This assumption is supported by the measurement of an increased LVEDV. Since this study is the first to investigate VV ECMO therapy in rats with a left ventricular pressure–volume catheter, these results cannot be compared to other studies.

This study revealed elevated concentrations of TNF-α during VV ECMO therapy in the rats. Although a high degree of data scattering cannot be ignored, significant differences were measured despite lung-protective ventilation. It has to be noted that the initial response of the innate immune system to injury and infection is reflected by TNF-α and IL-6. After activation, large amounts of TNF-α and IL-6 are released by monocytes, which results in systemic inflammatory response syndrome [28]. Many studies have demonstrated ECMO-induced inflammation during VA ECMO therapy in rats [8,11,12,13,15]. As studies of VV ECMO therapy in rats are either performed during ARDS or do not measure cytokines, our results cannot be compared to other studies [17,18,19,20,21]. A recent meta-analysis has shown that the levels of IL-6 and TNF-α are valuable prognostic indicators for patients with sepsis in the intensive care unit [29]. Contrary to our previous work with VA ECMO rat models, no differences regarding IL-6 and IL-10 were measured [8,13]. Further studies are needed to clarify if these differences are caused by the application of VV ECMO or other factors like lung-protective ventilation.

This study has some limitations. First, the results of animal studies cannot be directly transferred to humans. Nevertheless, the cardiopulmonary system of rats is comparable to that of humans. Second, contrary to clinical practice, the F_i_O_2mem_ was not set to 1.0. Nonetheless, sufficient oxygenation and decarboxylation were measured in our study. Third, the time course of the pO_2_ in the sham group indicates a reduced pulmonary function at the end of the experiments. This may represent the injury induced by the mechanical ventilation. It must be taken into account that the animals of the VV ECMO group received lung-protective ventilation. Nevertheless, increased levels of TNF-α were measured in the VV ECMO group. Fourth, the duration of VV ECMO therapy was very short compared to the clinical situation in the critical care unit. Nevertheless, hemodynamic changes were seen directly after the start of the ECMO. Furthermore, a “rat hour” is not equivalent to a “human hour” [30]. It has been shown that the genomic responses in various inflammatory conditions were 30–50 times faster in rodents (mice) than in humans [31]. In addition, the longer duration of ECMO therapy in rats is complicated by the repetitive blood withdrawal for blood gas analysis due to the limited blood volume of rats.

## 5. Conclusions

In summary, this study established a novel model of VV ECMO in rats with femoral drainage to answer further research questions. Blood flows of 90 mL/kg/min were achieved, and sufficient oxygenation and decarboxylation were demonstrated. Furthermore, hemodynamic changes during VV ECMO therapy, like increasing SV, CO, and LVEDV, were presented. Moreover, ECMO-induced inflammation was shown through increased concentrations of TNF-α. This model seems sufficient to answer research questions about topics such as intestinal microcirculation during VV ECMO therapy in further studies.

## Figures and Tables

**Table 1:** Results of the blood gas analysis.

		0 Min	30 Min	60 Min	90 Min	120 Min
pH	ECMO	7.38 [7.37–7.43]	7.39 [7.35–7.43]	7.40 [7.39–7.41]	7.39 [7.37–7.42]	7.40 [7.37–7.42]
	SHAM	7.38 [7.35–7.39]	7.36 [7.35–7.40]	7.40 [7.39–7.41]	7.37 [7.34–7.42]	7.36 [7.35–7.41]
Bic	ECMO	23.4 [22.7–24.1]	23.0 [22.0–23.1]	24.0 [23.3–24.9]	22.9 [22.0–24.2]	23.7 [23.1–24.0]
[mmol/L]	SHAM	22.9 [21.8–24.7]	23.2 [22.5–26.1]	23.8 [22.4–26.6]	23.4 [21.7–25.9]	23.2 [22.1–24.6]
BE	ECMO	−1.6 [−2.6–−0.9]	−1.7 [−3.0–−1.1]	−1.0 [−1.6–0.2]	−1.8 [−2.7–−0.3]	−1.1 [−1.6–−0.6]
	SHAM	−1.4 [−3.2–0.2]	−1.4 [−2.2–1.4]	−0.8 [−2.4–2.3]	−1.7 [−3–1.2]	−2.0 [−3.4–−0.2]
Lac **	ECMO	1.5 [1.0–1.6]	1.2 [1.1–1.4]	1.0 [0.9–1.2]	1.3 [1.1–1.5]	1.2 [1.0–1.3]
[mmol/L]	SHAM	1.5 [1.2–1.7]	1.0 [0.9–1.0]	0.9 [0.8–1.1]	0.9 [0.9–1.1]	0.9 [0.8–1.1]
Hb ***	ECMO	14.4 [14.1–14.8]	7.6 [7.4–7.8]	7.5 [7.3–8.0]	7.4 [6.8–7.6]	7.1 [6.8–7.5]
[g/dL]	SHAM	14.2 [13.8–15.0]	12.7 [12.0–14.5]	12.2 [11.5–13.2]	11.0 [9.9–11.7]	10.1 [9.6–11.0]
Glu *	ECMO	161 [140–172]	126 [121–131]	117 [112–122]	114 [111–117]	118 [111–124]
[mg/dL]	SHAM	166 [160–186]	139 [129–149]	129 [119–136]	121 [113–125]	115 [110–121]
Na	ECMO	144 [142–144]	144 [143–145]	145 [144–146]	146 [144–146]	145 [144–146]
[mmol/L]	SHAM	141 [141–145]	143 [141–144]	143 [142–144]	143 [142–145]	143 [142–145]
K **	ECMO	4.1 [3.9–4.3]	4.0 [3.8–4.1]	4.1 [3.9–4.1]	4.1 [ 3.9–4.3]	4.1 [4.0–4.3]
[mmol/L]	SHAM	4.2 [3.9–4.5]	4.5 [4.4–4.5]	4.4 [4.3–4.5]	4.3 [4.2–4.4]	4.3 [4.1–4.4]
Cl **	ECMO	109 [108–110]	112 [110–113]	112 [111–113]	113 [112–114]	113 [112–114]
[mmol/L]	SHAM	107 [106–109]	109 [107–111]	109 [107–111]	111 [109–112]	112 [110–113]

Data are presented as medians with 25th and 75th percentiles. The asterisks denote the degree of statistical significance (* *p* < 0.05; ** *p* < 0.01; *** *p* < 0.001). Abbreviations: BE = base excess; Bic = bicarbonate; Cl = chloride; Glu = glucose; Hb = hemoglobin; K = potassium; Lac = lactate; Na = sodium.

## Data Availability

The original contributions presented in the study are included in the article; further inquiries can be directed to the corresponding author.
